# A second type of N7-guanine RNA cap methyltransferase in an unusual locus of a large RNA virus genome

**DOI:** 10.1093/nar/gkac876

**Published:** 2022-10-21

**Authors:** Ashleigh Shannon, Bhawna Sama, Pierre Gauffre, Théo Guez, Françoise Debart, Jean-Jacques Vasseur, Etienne Decroly, Bruno Canard, François Ferron

**Affiliations:** Aix-Marseille Université and CNRS, Laboratoire Architecture et Fonction des Macromolécules Biologiques, UMR 7257, 13009, Marseille, France; Aix-Marseille Université and CNRS, Laboratoire Architecture et Fonction des Macromolécules Biologiques, UMR 7257, 13009, Marseille, France; Aix-Marseille Université and CNRS, Laboratoire Architecture et Fonction des Macromolécules Biologiques, UMR 7257, 13009, Marseille, France; IBMM, University of Montpellier, CNRS, ENSCM, Montpellier, France; IBMM, University of Montpellier, CNRS, ENSCM, Montpellier, France; IBMM, University of Montpellier, CNRS, ENSCM, Montpellier, France; Aix-Marseille Université and CNRS, Laboratoire Architecture et Fonction des Macromolécules Biologiques, UMR 7257, 13009, Marseille, France; Aix-Marseille Université and CNRS, Laboratoire Architecture et Fonction des Macromolécules Biologiques, UMR 7257, 13009, Marseille, France; European Virus Bioinformatics Center, Leutragraben 1, 07743 Jena, Germany; Aix-Marseille Université and CNRS, Laboratoire Architecture et Fonction des Macromolécules Biologiques, UMR 7257, 13009, Marseille, France; European Virus Bioinformatics Center, Leutragraben 1, 07743 Jena, Germany

## Abstract

The order *Nidovirales* is a diverse group of (+)RNA viruses, with a common genome organization and conserved set of replicative and editing enzymes. In particular, RNA methyltransferases play a central role in mRNA stability and immune escape. However, their presence and distribution in different *Nidovirales* families is not homogeneous. In *Coronaviridae*, the best characterized family, two distinct methytransferases perform methylation of the N7-guanine and 2′-OH of the RNA-cap to generate a cap-1 structure (m7GpppNm). The genes of both of these enzymes are located in the ORF1b genomic region. While 2′-*O*-MTases can be identified for most other families based on conservation of both sequence motifs and genetic loci, identification of the N7-guanine methyltransferase has proved more challenging. Recently, we identified a putative N7-MTase domain in the ORF1a region (N7-MT-1a) of certain members of the large genome *Tobaniviridae* family. Here, we demonstrate that this domain indeed harbors N7-specific methyltransferase activity. We present its structure as the first N7-specific Rossmann-fold (RF) MTase identified for (+)RNA viruses, making it remarkably different from that of the known *Coronaviridae* ORF1b N7-MTase gene. We discuss the evolutionary implications of such an appearance in this unexpected location in the genome, which introduces a split-off in the classification of *Tobaniviridae*.

## INTRODUCTION

The *Nidovirales* order encompasses a remarkably diverse group of enveloped, positive-sense RNA viruses with genome sizes ranging from ∼11 to 41 kb. The order is currently classified into 14 families, with members infecting a range of hosts (eight vertebrate and six invertebrate families) (1,2). At one end of the size spectrum are the small-genome *Arteriviridae* members, with genomic RNAs ranging in size from ∼11 to 15 kb. At the other end of the spectrum, *Coronaviridae* genomes reach >32 kb in size, topped only by a few members of the (currently) under populated families such as the *Mononiviridae* (>41 kb). Despite these significant size-differences, these viruses are classified together based on their relatively conserved genome organization and mRNA transcriptional scheme. In the majority of nidoviruses, the first two-thirds of the genome contains two large overlapping open reading frames (ORF1a and 1b). Translation of the ORFs yields two polyproteins (pp1a and pp1ab) containing between 12 and 16 non-structural proteins involved in viral replication and transcription. The 3′ end of the genome codes for a nested set of subgenomic mRNAs (sg mRNAs) directing the synthesis of structural and accessory proteins. The name of the order comes from *Nido* (*latin*: nest), which refers to this common ‘nested’ gene expression pattern allowing differential expression levels of non-structural versus structural and accessory proteins (3).

Due to their unusual size, large *Nidovirales* RNA genomes and transcripts need both genetic and biophysical stability in order to faithfully transmit and express their genetic content. Genetic stability is generally ensured by the replication/transcription complex (RTC), composed of a core RNA-dependent RNA polymerase (nsp12) flanked with processivity factors nsp7 and nsp8 (4). The RTC also recruits nsp14, a 3'-to-5′ exonuclease (ExoN) which corrects replication errors (5). This mismatch repair system, unique to large genome nidoviruses, ensures accurate replication and transfer of genomic information, and is thus essential for genetic stability (6).

Many +RNA viruses have additionally evolved mechanisms to protect their RNAs by the incorporation of a 5′ cap. As is the case for eukaryotic mRNAs, this 5′ cap allows recognition and translation of viral mRNA by host ribosomes, and additionally protects transcripts from 5′-to-3′ exonucleolytic degradation. Viral RNA capping is thus essential not only for genome stability and translation, but also for innate-immune escape since the viral RNA cap cannot be distinguished from its cellular counterpart (7–11).

For the nidoviruses, the presence of an RNA cap has been reported for three distinct viruses within the order (12–15), but high-resolution structural analysis of nidovirus cap structures is still lacking. Further indication of the presence of a 5′ type-1 cap is indirectly provided by the identification of key enzymes required in the conventional, eukaryotic capping pathway (16). Among these enzymes are i) an RNA triphosphatase (RTPase), which is responsible for hydrolysis of the 5′-triphosphate of the viral RNA into a 5′-diphosphate, ii) a guanylyltransferase (GTase), which covalently transfers a GMP moiety (originating from GTP) to the 5′-diphosphate, generating a 5′ṇ5′ triphosphate bridge (GpppN) and iii) one or two methyltransferases (MTases), that subsequently methylate the cap using *S*-adenosyl-methionine (AdoMet or SAM) as the methyl donor, converting it to *S*-adenosyl-homocysteine (AdoHcy or SAH). Methylation generally occurs in a sequential order, with the N7-guanine methylated first for generation of the ^m^GpppN-RNA (type-0 cap), followed by the 2′-hydroxyl position of the first transcribed nucleotide, yielding ^m^GpppN_m_-RNA (type-1 cap).

Most large RNA viruses (>17 kb) of positive genome polarity generally encode at least one MTase gene, and it has been proposed that the correlation between RNA genome size and presence of an MTase reflects a beneficial coevolution (17). Five distinct structural classes of enzymes that catalyze SAM-dependent methylation have been described, with most viral MTases (along with most DNA methyltransferases), falling into the largest, Class I group (18). Class I MTases are characterized by the presence of a Rossmann-fold (RF) domain, a super-secondary structure adopted by a diverse group of dinucleotide binding enzymes. It is generally characterized by an alternating βαβ architecutre, made up of of a central β-sheet with up to seven β-strands, surrounded by α-helices. The glycine-rich motif I (GxGxG) follows the first β-strand, and is involved in SAM binding.

For *Coronaviridae*, most of the enzymes required in this capping pathway have now been identified, including the N7-guanine and 2′-*O* MTases, which reside in nsp14 and nsp16, respectively (19–21). The RTPase resides in nsp13 (22) while more recently, the GTase activity was attributed to the nidovirus RdRp-associated nucleotidyl transferase (NiRAN) domain, located at the N-terminus of nsp12 (23–26). However, the precise mechanism of action of this enzyme, including the requirement for viral cofactors and specific substrate requirements, is still somewhat speculative. For most other nidoviral families, the capping pathway and enzymes have been poorly characterized. Capping enzymes which are homologous to those in the *C*oronaviridae genome have been identified for several families (19–22,25,27,28). In contrast, for the the small-genome *Arteriviridae* family, none of the enzymes required in the capping pathway appear to be present, suggesting that the presence of an RNA cap is not conserved throughout the entire order.

Recently, we performed a large-scale genomic analysis of the order *Nidovirales* in order to clarify the presence of MTase domains across different families (29). The 2′*O* MTase can be clearly identified for most of these viruses based on a conserved genomic location (encoded at the end of ORF1b), and can be further characterized by their RF structure and K–D–K–E catalytic tetrad. Unlike 2′*O*-MTases, RF N7-MTases lack a specific catalytic signature sequence. As such, determining their methylation-site specificity is more challenging. Furthermore, the N7-MTase domain residing in nsp14 of CoVs shows very limited sequence conservation with other N7-MTases, and lacks the βαβ architecture characteristic of RF enzymes. While nsp14-like, non-RF N7-MTases can also be identified for several other nidoviruses, this enzyme appears to be lacking entirely for many of the families. Interestingly however, we and others have identified a putative N7-MTase in pp1a (N7-MT-1a) of several members of the large-genome *Tobaniviridae* family (29–31).

Here we confirm that this domain is indeed the missing N7-MTase. We present its crystal structure at 1.7 Å resolution, revealing that it adopts a canonical RF secondary structure. This makes it the first N7-specific RF MTase identified for any positive-stranded RNA virus to date. Its closest homologues are the N7-MTases of the microsporidian parasite *Encephalitozoon cuniculi*, humans and two large DNA viruses—despite extremely limited sequence identity with any of these MTases. In regards to the genomic position, sequence and predicted secondary structure, this domain is remarkably different from the known nidovirus nsp14 N7-MTase gene, illustrating an outstanding genetic diversity within a single order of viruses. Intriguingly, this domain only appears to be present in non-mammalian infecting (fish or reptile host) tobanivirus members (29). The characterization of this new N7-MTase will not only aid with further clarification within this viral order, but will assist in the identification and assignment of MTases across all domains of life.

## MATERIALS AND METHODS

### Plasmid construction

An *Escherichia coli* codon optimized sequence for the region spanning Met^1333^ to Ile^1668^ of White Bream virus (WBV, NCBI Reference YP_803214.1) was synthesized by GenScript, with EcoRI and HindIII restriction enzyme sequences flanking the 5′ and 3′ ends respectively. The synthesized gene was subcloned into a pQE30 expression vector, under the control of a T5 promoter, incorporating a 5′ hexahistidine sequence. Deletion PCR mutagenesis with outward facing primers was subsequently performed using Q5 site-directed mutagenesis (New England Biolabs), to delete regions from the 5′ end region. Final recombinant plasmids contained domains starting from Met^1333^, Thr^1342^, Thr^1350^, Ser^1366^, Ala^1358^, Asp^1374^ or Ser^1389^, spanning to Ile^1668^. A final C-terminal truncation was additionally performed for expression of a 260 amino acid (∼30 kDa) domain spanning from Asp^1374^–Asp^1633^. This construct was subsequently used for site-directed mutagenesis using outwards facing, overlapping primers and High Fidelity Phusion Polymerase (NEB).

### Protein expression and purification

Protein was expressed in C2523 (pLacI) *E. coli* overnight at 18°C from cultures induced with 0.4mM isopropyl-β-d-thiogalactopyranoside and 2% ethanol once the OD_600_ reaction 0.8. Bacterial pellets were resuspended in lysis buffer (20 mM HEPES pH 7.5, 500 mM NaCl, 10% glycerol, 2 mM BME, 1 mg/ml lysozyme, 20 μg/ml DNase, 1 mM PMSF), incubated for 30 min before lysis via sonication (amplitude 40%, 2 s on, 2 s off for 1 min). The soluble lysate was purified with affinity chromatography using HisPur Cobalt resin (Thermo Scientific). The bound lysate was washed with 2 column volumes wash buffer (20 mM HEPES pH 7.5, 500 mM NaCl, 10% glycerol, 20 mM imidazole, 2 mM BME) and eluted with 150 mM imidazole (20 mM HEPES pH 7.5, 300 mM NaCl, 10% glycerol, 150 mM imidazole, 2 mM BME). The purified protein was subsequently concentrated and loaded onto a Superdex 200 gel filtration column (GE Healthcare), eluted in gel filtration buffer (20 mM HEPES pH 7.5, 150 mM NaCl, 10% glycerol, 2.5 mM TCEP).

### Synthesis of RNA substrates

RNA synthesis was previously described in (32). RNA sequences were chemically synthesized on solid support using an ABI 394 oligonucleotides synthesizer. After RNA elongation with 2′-*O*-pivaloyloxymethyl phosphoramidite ribonucleotides (33) and 2′-*O*-methyl phosphoramidite ribonucleotides (Chemgenes, USA), the 5′-hydroxyl group was phosphorylated and the resulting H-phosphonate derivative was oxidized and activated into a phosphoroimidazolidate derivative (34) to react with guanosine diphosphate yielding Gppp-RNA (35). After deprotection and release from the solid support, GpppRNAs were purified by IEX-HPLC and they were characterized by MALDI-TOF spectrometry. N7-methylation of the purified GpppRNA was performed enzymatically using N7-hMTase to give mGppp-RNA (35). All sequences used for analysis are listed in Supplementary Table S1.

### Filter binding assays for methylation activity

Methylation activity was performed using filter binding assays, with reactions prepared in 96-well plates. For standard assays, final buffer conditions were 40 mM Tris pH 8, 1 mM DTT, 0.7 μM RNA, 1.9 μM cold AdoMet, supplemented with 0.1 μM ^3^H-AdoMet. Initial reactions to assess potential activity were run with final enzyme concentrations ranging from 10 nM – 500 nM, with subsequent assays run with 10–500 nM enzyme, as stated in figure legends. Reactions were quenched at various time points by diluting five-fold in a solution of 200 μM AdoHcy, then further diluted to a 300 ul total volume in ice-cold H2O. A Filtermat Harvester (Packard Instruments) was used to transfer the reactions to 8 × 12 filtermats (GF/C, Perkin Elmer) coated with a 0.3% (w/v) solution of polyethylenimine (PEI, Sigma-Aldrich) for RNA binding. Filtermats were washed twice with 10 mM ammonium formate pH 8.0, twice with water and once with ethanol, prior to soaking in liquid scintillation fluid. RNA methylated with ^3^H-AdoMet was measured using a Wallace MicroBeta TriLux Liquid Scintillation Counter (Perking Elmer).

### Thin layer chromatography (TLC)

Triphosphorylated RNA (pppG-RNA_12_ or pppA-RNA_12_, 10 μM) were capped using the vaccinia virus capping enzyme (New England Biolabs) in the presence of 2 μCi α-^32^P-GTP (Perkin Elmer) to yield radiolabeled G*pppN capped RNA (where * represents the radioactive α-^32^P). Duplicate reactions were run in the presence of 0.1 mM AdoMet, for creation of N7-guanine methylated cap controls (^m^G*ppppN). Additional reactions were additionally prepared using pppN_m_-RNA_12_ (pre-methylated at the 2′*O* position), in the presence or absence of AdoMet, for generation of G*pppN_m_ and ^m^G*pppN_m_ cap controls. Capped RNA was incubated with 1 μM (final concentration) of the purified WB MTase enzyme in a final reaction containing 40 mM Tris, pH 8, 40 μM AdoMet, and 1 μM capped RNA. RNA was digested with Nuclease P1 (New England Biolabs) for 3–4 h at 37°C, and loaded onto polyethylenimine cellulose TLC sheets (Macherey Nagel), along with cap controls. Samples were separated using 0.65 M LiCl as mobile phase. TLC sheets were exposed and visualized using using an Amersham Typhoon BiomolecularImager (GE Healthcare).

### Crystallization and structure determination

Crystallization conditions were initially identified using the sitting-drop vapor diffusion method using INDEX and PEGII 96 reagent screens (Hampton Research). Conditions were optimized for the WBV 260 construct (WBV-260, Asp^1374^–Asp^1633^), mixed with AdoMet (New England Biosciences) at a 1:1.2 ratio prior to crystallization. All crystals were grown at 293.15 K, using a 1:1 ratio of protein (8 mg/mL) to precipitant solution (0.1 M Tris pH 7.7, 20.3% PEG 3350, 0.2 M MgCl and 10% isopropanol), and grew in around 5 days as long needles/urchins. The presence of isopropanol drastically improved the size and diffraction of crystals. In our attempt to phase, several crystals were soaked in Tantalum Bromide and harvested while green, according to manufacturer protocol (Jena Bioscience). All crystals were cryo- protected with reservoir solution supplemented with 20% PEG400, and flash-frozen in liquid nitrogen at 100 K. Data collection and diffraction data were collected at Soleil synchrotron, from several crystals. Original native and Sulfure SAD data set were collected on Proxima2, under the assumption that we could use experimental phasing to solve the structure. Further Sulfure SAD and Tantalum SAD data were collected on Proxima1. Data sets were processed individually and analysed with autoPROC toolbox (36). For crystals soaked in Tantalum Bromide, we obtained crystals in C2 and P21 space groups, however no significant anomalous signal was detected. Distant homologous structures failed to give phased solution, ultimately, a model was generated using AlphaFold2 (37) and structure was solved by molecular replacement using PHASER (38). The first model was rebuild in COOT (39). and refined at 2.63 Å using PHENIX (40). That model was used as solution for molecular replacement using PHASER and structure was refined using BUSTER (41,42) at 1.66 Å. Both structures were checked and confirmed to have good stereochemistry according to MOLPROBITY (43). Data collection and refinement statistics are listed in Table 1.

**Table 1.** Data collection and refinement statistics

	**White Bream N7-MTase** Proxima 2 | PDB: 7Z05	**White Bream N7-MTase** Proxima 1 | PDB : 7Z2J
**Wavelength**	0.992	1.255
**Resolution range**	44.93–2.33 (2.41–2.33)	39.07–1.66 (1.72–1.66)
**Space group**	*C* 1 2 1	*P* 1 21 1
**Unit cell**	105.22, 49.787, 53.93	41.58, 49.02, 53.76
	90.00, 97.72, 90.00	90.00, 110.04, 90.00
**Total reflections**	80 601 (7962)	159 906 (14 462)
**Unique reflections**	11 969 (1168)	24 131 (2383)
**Multiplicity**	6.7 (6.8)	6.6 (6.1)
**Completeness (%)**	99.62 (99.57)	99.81 (99.04)
**Mean *I*/sigma(*I*)**	11.93 (2.28)	16.16 (2.41)
**Wilson *B*-factor**	39.11	30.35
***R*-merge**	0.115 (1.08)	0.056 (0.64)
***R*-meas**	0.12 (1.17)	0.061 (0.70)
***R*-pim**	0.04817 (0.44)	0.02 (0.28)
**CC1/2**	0.99 (0.874)	0.99 (0.91)
CC*	0.99 (0.966)	1 (0.97)
**Reflections used in refinement**	11942 (1164)	24129 (2383)
**Reflections used for *R*-free**	582 (52)	1168 (121)
***R*-work**	0.23 (0.36)	0.19 (0.38)
***R*-free**	0.24 (0.35)	0.20 (0.42)
**CC(work)**	0.94 (0.85)	0.94 (0.32)
**CC(free)**	0.94 (0.86)	0.93 (0.37)
**Number of non-hydrogen atoms**	2013	1835
Macromolecules	1924	1780
Ligands	15	0
Solvent	74	55
**Protein residues**	240	228
RMS(bonds)	0.012	0.014
RMS(angles)	1.64	1.93
Ramachandran favored (%)	92.83	97.33
Ramachandran allowed (%)	6.33	2.22
Ramachandran outliers (%)	0.84	0.44
Rotamer outliers (%)	6.91	3.03
Clashscore	12.40	1.13
**Average *B*-factor**	55.43	38.89
Macromolecules	55.55	38.58
Ligands	60.03	
Solvent	51.57	49.14

Statistics for the highest-resolution shell are shown in parentheses.

### Structural analysis and sequence comparison of N7-MTase domain

Homologous structure searches using the DALI and PDBe-fold servers (44,45) retrieved the N7-MTases of the large DNA virus, African Swine Fever virus (ASFV, PDB 7D8U), *E. cuniculi* (PDB 1Z3C), human (PDB 3BGV) and Vaccinia virus (PDB 2VDW) as the closest homologues (sequence identity between 14 and 16%, *z*-score ranging from 17 to 20). Structural superimpositions of these structures was done manually using UCSF CHIMERA (46). From the structural superimposition, we generated a multiple sequence alignment (MSA) and visualized with each corresponding secondary structure in ESPript (47). Secondary structure analysis of WB N7-MTase was done using ENDscript (47). For motif identification, each sequence from the structural MSA was used as a BLAST input search against NR_50_1Nov (MPI TOOLKIT) (48). All retrieved sequences were merged in SEAVIEW (49) and sequences with 90% identity and above were discarded to avoid cross-referencing. The 102 remaining sequences were aligned according to the structural MSA profile. Visualization of the motifs was done using WebLogo (50).

### Sequence comparison of Tobaniviridae family members

The presence/absence of the N7-MT-1a domain was compared via MSA using Clustal Omega (51) on SeaView for the 23 *Tobaniviridae* members (Supplementary Table S2), as recognised under the latest ICTV proposal (https://ictv.global/ICTV/proposals/2021.005S.R.Nidovirales.zip). The MSA was then manually modified (respecting amino acid chemistry and with no random gap introduction) based on the most conserved motifs/residues, determined from the extended structural MSA profile. Secondary structure prediction using PHD (52) were used to refine the alignment, in particular the conservation of the α helix and β strand secondary elements, corresponding to the characteristic β1–α2–β2 RF signature fold. The final alignment was visualized together with secondary structures of WBV, CSV, HpoToV_33749, VCSTV-A, MVNV, and SerTV-C18 using ESPript. The specific tobani- N7-MTase key motifs were visualized using WebLogo.

## RESULTS

### Expression of a 311 amino acid domain of the putative N7-MTase in White Bream virus yields a soluble, active enzyme

**Figure 1.**
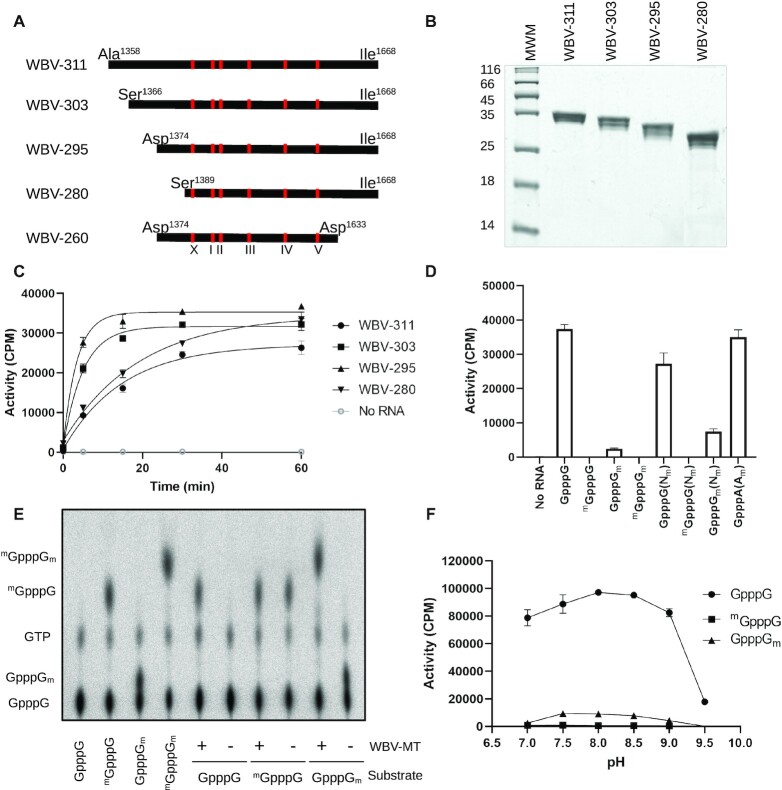
Cap-dependent methyltransferase activity of the WBV N7-MT-1a domain. For (C), (D) and (F), the transfer of tritiated methyl groups from AdoMet to capped RNA substrates (Table S1) was measured through filter binding assays and shown as counts per minute (CPM). (**A**) Schematic of the different constructs of N7-MT-1a. Limits indicate the starting amino acid and numbering in respect to 1a polyprotein, with approximate location of conserved domains (I, II, III, IV, V and X) shown. (**B**) SDS-PAGE analysis of different constructs. (**C**) Timecourse comparing the activity of different lengths of the putative N7-MT-1a domain from WBV. For the WBV-280 construct, final enzyme concentration in assays was 500 nM. For all other constructs, final enzyme concentration was 20 nM. (**D**) Methylation of different capped RNA_13_ substrates premethylated at the guanine N7 position (^m^GpppN) or 2′*O* ribose groups of the first nucleotide (Gppp*N*_m_), or internal residues of the RNA (Gppp*N*(*N*_m_)), where *N* is all residues. Activity represents single, 30 minute timepoint measured for the WBV-295 product, and is shown as the mean ± SD (*n* = 3). (**E**) Thin-layer chromatography analysis showing migration of differently methylated GpppG cap structures. The first four lanes show migration of cap controls, prepared with the commercial, vaccinia virus N7-MTase. The remainder of gel shows modification of different capped RNAs (substrate) in the presence (+) or absence (−) of the WBV N7-MT-1a. (**F**) Methylation activity of the WBV-295 product at different pH on different capped RNA_9_ substrates. The plotted values represent a single, 30 min timepoint shown as the mean ± SD (*n* = 3).

To confirm the recent identification of a putative N7-MTase domain in the pp1a of non-mammalian tobaniviruses, we first performed expression screens for one of the members of this family, White Bream virus (WBV). Alignments with the vaccinia virus D1 subunit N7-MTase (VV-D1) and other confirmed N7-MTases reveals several conserved domains, however the the natural N- and C-terminal borders, and importance of these regions for activity was unclear. As such, several lengths of the putative MTase, ranging from 260 to 336 amino acids in length were expressed and purified. All expressed domains include the five conserved motifs identified by structural alignment with the vaccinia virus D1 subunit N7-MTase (VV-D1). High yields of soluble protein (∼20–30 mg/l culture) can be obtained from a 311 amino acid domain spanning residues Ala^1358^–Ile^1668^ (WBV-311) (Figure 1A). Gene constructs longer than this were insoluble. Expression of three N-terminally truncated domains, starting at Ser^1366^ (WBV-303), Asp^1374^ (WBV-295) or Ser^1389^ (WBV-280) produces similar yields of soluble proteins. However, all four purified expression products appear as a double-band at their approximate molecular weights (31–34 kDa) when analyzed by SDS-PAGE (Figure 1B), suggesting that the protein is partially cleaved at the C-terminus during expression.

The soluble protein products were tested by filter binding assays (FBA) for their ability to methylate a short (13-mer) GpppG-capped RNA substrate using radioactive ^3^H-AdoMet as the methyl donor. High levels of methyltransfer are observed for three out of the four soluble proteins, with no activity for controls lacking either RNA or protein (Figure 1C). WBV-295 is the most active product, and was subsequently selected to further characterize MTase activity. The shortest product (WBV-280) is >100-fold less active (25-fold higher enzyme concentration used in assay), indicating that the 16 N-terminal residues (Asp^1374^–Ser^1383^) of WBV-295 are functionally important. These results confirm that the coding sequence encompassed by Asp^1374^–Ile^1668^ in the ORF1a of WBV harbors methyltransferase activity.

### The WBV-MTase harbors N7-guanine specific activity

Methylation specificity was determined using FBA with a series of capped RNAs (RNA_13_, Supplementary Table S1), pre-methylated at different positions of the cap structure (^m^GpppN, GpppN_m_, ^m^GpppN_m_) (Figure 1D). High levels of methylation are observed for any substrate with an unmethylated cap structure (Gppp*N*). The first nucleotide of the WBV genome is a G, however both GpppG and GpppA capped RNA substrates are methylated equally well (Figure 1D). Likewise, no difference in activity is observed when using a different RNA (GpppG-RNA_9_, Supplementary Table S1), indicating that neither the identity of the first nucleotide nor the RNA sequence influences substrate recognition and/or methylation (Supplementary Figure S1a). Activity is blocked by pre-methylation at the N7-guanine position (^m^GpppG-RNA, Figure 1D). Methylation is also drastically reduced for substrates carrying a 2′*O*-methylated ribose (Figure 1D). These results suggest that the WBV MTase specifically targets the N7-guanine site, and that N7-methylation precedes ribose 2′*O*-methylation, as is the case for other viruses (21,53). To confirm that the methylation activity was specifically targeted to the N7 position of the guanine, and not the less conventional N3 position, we performed TLC migration analysis using P^32^ radiolabelled α-GTP as the cap donor. Following incubation of either GpppG- or GpppA-RNA with the WBV MTase, caps migrate to the expected ^m7^GpppN position (Figure 1E, Supplementary Figure S1b). No change in migration is observed for caps pre-methylated at the N7 position, while incubation with GpppN_m_-RNA yields products that migrated to the ^m7^GpppG_m_ cap-1 structure, consistent with N7-guanine specificity. In contrast to the FBA, 100% of the GpppG_m_ substrate is converted to the ^m7^GpppG_m_ when analyzed via TLC. This was attributed to the higher concentrations of enzyme and substrate used for TLC analysis, in addition to the longer incubation times. FBA analysis with increasing enzyme concentrations, similar to that used in TLC, confirmed this to be the case (Supplementary Figure S1c, d). We therefore conclude that while an unmethylated cap structure (GpppN) is the preferred substrate for N7-guanine methylation, GpppG_m_ capped RNAs are also able to be methylated, albeit at a slower rate.

For bi-functional MTases, the preference for N7 or 2′*O* methylation is pH-dependent (32,54). For WBV, N7-methylation is optimal at pH 8–8.5, with no 2′*O* methylation at any pH, suggesting that this domain does not have dual functionality (Figure 1F). Some MTases also harbor internal 2′O methylation activity (32). For the WBV-MTase, no activity is observed with ^m^GpppG_m_-RNA substrates (Figure 1D). In addition, capped RNAs containing partial, or complete internal 2′*O*-ribose methylations (Gppp*N*(*N*_m_)) are highly methylated, confirming that this MTase does not target internal residues (Figure 1D). The SARS-CoV nsp14 N7-MTase can also utilize GTP as an acceptor substrate for methylation (55). On the contrary, the WBV N7-MTase is unable to methylate GTP only (data not shown). The WBV MTase is therefore RNA Cap-guanine specific, with a strong preference for methylation of an open (GpppN) cap structure.

### Structural overview of the *Tobaniviridae* N7-MT-1a

Crystals of the WBV N7-MT-1a were obtained using the WBV-295 construct, however they grew as thin needles that diffracted poorly (∼6 Å). A C-terminally truncated construct (Asp^1374^–Asp^1633^, WBV-260) yielded a clean product of expected molecular weight, possessing activity levels equivalent to WBV-295 (Supplementary Figure S2). The WBV-260 protein crystallized in two different space groups, C2 and *P*2_1_, with one monomer per asymmetric unit, diffracting to 2.6 and 1.7 Å, respectively (Table 1). The difference is likely due to soaking with tantalum bromide (C2 crystals), creating an additional constraint for the crystal. The two structures are almost identical, with the exception of the last flexible C-terminal helix that is not visible in the Tantalum-soaked crystal. AdoHcy, the by-product of the methylation reaction, is found in the active-site of both structures.

**Figure 2.**
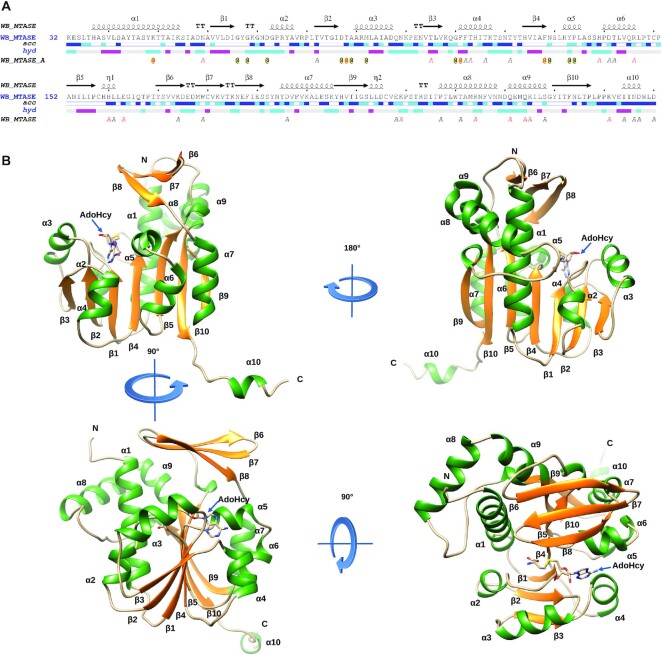
Sequence and structure of WBV N7-MT-1a. (**A**) Sequence of the resolved N7-MT-1a domain with the corresponding structure elements shown above, and solvent accessibility (acc; blue-exposed, cyan-intermediate, white-buried) and residues relative hydrophobicity (hyd; pink-hydrophobic, grey-intermediate, cyan-hydrophilic). Residues interacting with SAH are marked with an @. (**B**) Ribbon model of N7-MT-1a domain colored by secondary structures: loop (tan), α-helix (green), β-strand (gold). Secondary structures are labeled according to (A).

The central core of the WBV N7-MT-1a adopts a canonical Rossmann-fold (RF), with an alternating βαβ structural motif, characteristic of the class I family of MTases. This core structure is formed by a central, seven-stranded β-sheet (ordered β3, β2, β1, β4, β5, β10, β9), flanked by α-helices on each side (α1, α2, α3 on one side, and α4, α5/α6 and α7 on the other). The final β-strand (β10) is inserted in an anti-parallel orientation between the fifth and seventh strands (β5 and β9) of the β-sheet. In addition to this central fold, two other structural features are present; a flexible ‘3β-flap’ domain comprised of a short α-helical loop followed by three anti-parallel β-strands (β1 and β6–β8) and an α-helical bundle (β2, α8 and α9) wrapping around α1, herein referred to as the α-bundle region (Figure 2).

### Structural comparison with identified homologs

A structure similarity search retrieved the N7-MTase of the large DNA virus, African Swine Fever virus (ASFV, PDB 7D8U) as the closest homolog, with 16% sequence identity (*z*-score of 20.1 and a 2.6 Å rmsd) (56). Despite low sequence similarity (<15%) the N7-MTases from the microsporidian parasite *Encephalitozoon cuniculi*, Ecm1 (PDB ID: 1Z3C, 2.45 Å rmsd), VV-D1 (PDB ID: 2DVW and 4CKB, rmsd of 2.45 Å) and Hcm1 from humans (PDB ID: 5E8J, 2.3 Å rmsd) were additionally identified as potential homologues (Supplementary Figure S3). Superimposition shows the most significant structural variations are observed in the N-terminal domain, as well as in the 3β-flap and α-bundle regions.

The first 22 residues of the WBV N7-MT-1a are unresolved in the structure. This region is important for the methylation activity, as the WBV-280 construct (lacking residues Asp1 to Ser16) is > 100-fold less active than its longer counterpart, WBV-295 (Figure 1C). Comparatively, the N-terminal 40 amino acids (aa) are also missing from the Ecm1 structure, with at least 10 of these residues known to be important for activity (PDB ID: 1Z3C) (57). The structure of the Vaccinia virus D1 N7-MTase shows that the equivalent region (residues 545–560), folds over the AdoMet binding site, directly contributing to MTase activity (PDB ID: 2VDW). In contrast, the N-terminus of ASFV forms a β-strand, which does not contribute to the active-site, but rather stabilizes the crystal packing. Together, these results suggest that the N-terminal domains of these N7-MTases are highly flexible, yet important for catalytic activity and/or structural stability.

A comparison of the 3β-flap domains of these five MTases shows the WBV N7-MT-1a flap-structure is most similar to that of Ecm1, which is also formed by an α-helix and three-stranded antiparallel β-sheet (Supplementary Figure S3). Nevertheless, the WBV N7-MT-1a domain is more compact, as both the ŋ1 helix and the loop between the first two β-strands are shorter. Likewise, the human, ASFV and vaccinia virus N7-MTases also contain longer helix and loop elements, as well as a fourth antiparallel ${\rm{\beta }}$-strand, making their flap domains even larger.

The α-bundle domain is the most structurally diverse region of the MTase. While all related MTases possess a similar domain between β9 and β10 of the central β-sheet, the length, position, and number of α-helices vary substantially between these structures. The human N7-MTase contains an additional insertion in the α-bundle region (referred to as the lobe), which is absent in other structures, including WBV N7-MT-1a. The observed variability of the 3β-flap and α-bundle region is indicative of their dynamic role in the accommodation and stabilization of the RNA GpppG-cap structure during cap 0 synthesis and as such are structural markers of the N7-MTase fold.

**Figure 3.**
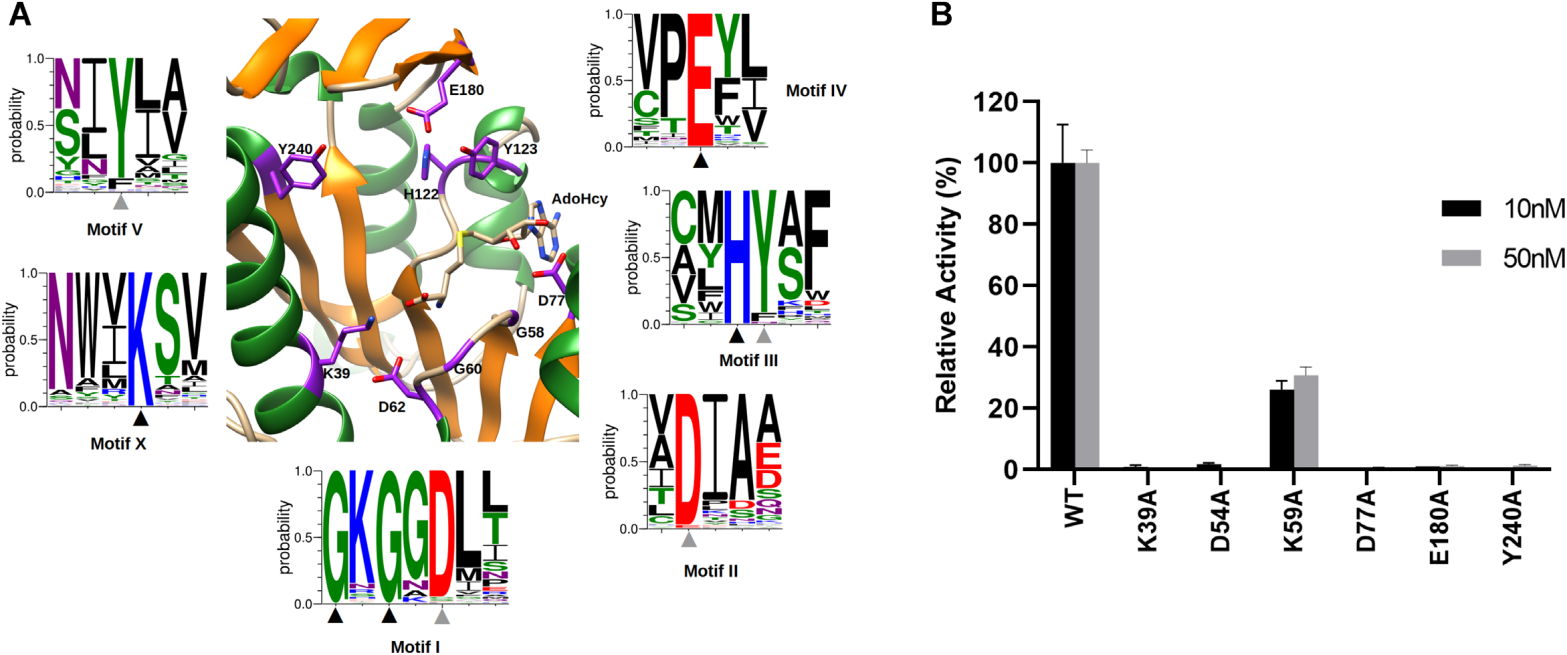
(**A**) Stuctural core conservation analysis. Central, catalytic site of the WBV N7-MT-1a in ribbons (same color code as in Figure 2) with side-chains of key residues shown in purple. WebLogo analysis of the six conserved motifs are shown around the structure, based on a structural MSA with other N7-guanine specific MTases. Key residues are indicated with a triangle (black if identical, grey if similar). (**B**) Activity of various WMV-260 mutants. The transfer of tritiated methyl groups from AdoMet to GpppG-RNA_13_ was measured through filter binding assays. Activity represents single, 30 min timepoint at two enzyme concentrations, and is shown as the mean ± SD relative to the activity of the wild-type enzyme (*n* = 3).

A sequence homology search using the structural alignment of the five N7-MTases retrieved a pool of 120 diverse sequences, of which 6 conserved sequence motifs involved either in AdoMet binding and catalytic transfer (motifs X, I, III, IV) or in the GpppG-cap structure binding (motifs III, IV, V) can be defined (Figure 3, discussed below).

### AdoMet/AdoHcy active-site pocket

**Figure 4.**
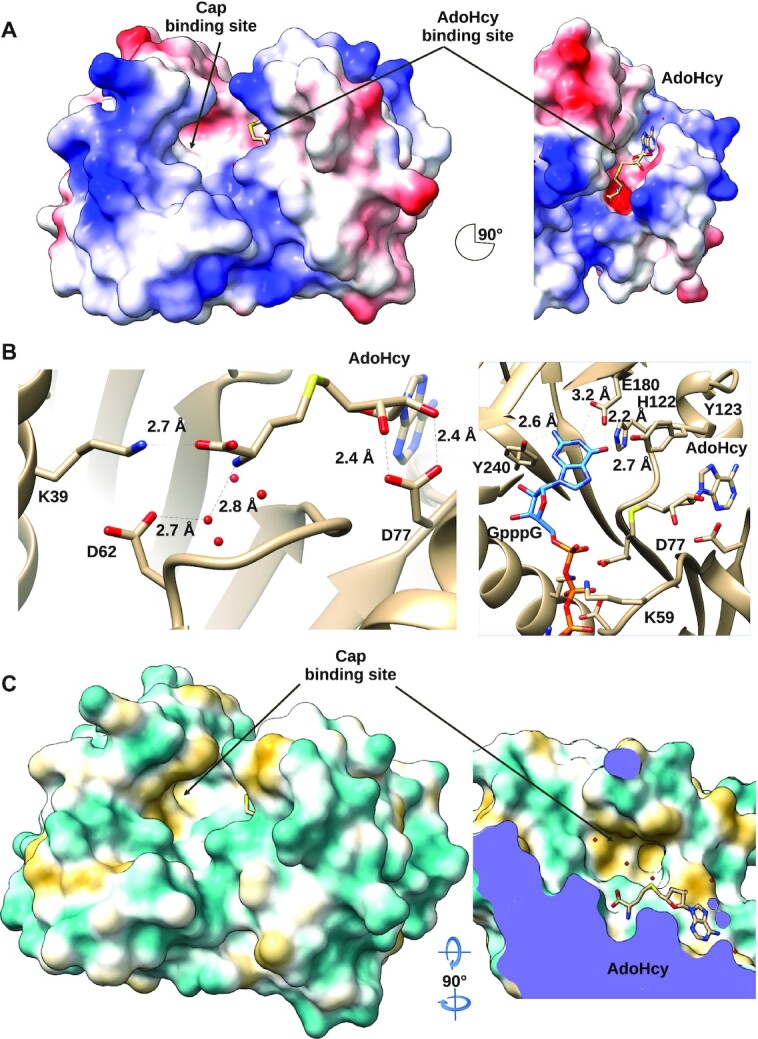
(**A**) Electrostatic surface potential of WBV N7-MT-1a, calculated by Adaptive Poisson–Boltzmann Solver, from –10 (red) to +10 (blue) kT/e. Left panel shows the cap binding site while the right panel is rotated 90° and shows the AdoHcy in AdoMet binding site. (**B**) Left panel: AdoMet binding site, with conserved residues shown in sticks, showing interactions with AdoHcy. Right panel: Predicted stabilization of GpppG, docked from PDB:1RI2 in WBV N7-MT-1a cap binding site. (**C**) Hydrophobic surface representation of WBV N7-MT-1a (yellow is hydrophobic, cyan is aliphatic). Left panel shows the cap binding site. The right panel is rotated 90° (on x and y axes) and sliced up to the AdoMet binding site.

As with other, class I MTases, AdoHcy binds in a deep, predominantly negatively charged groove (Figure 4A), formed by the loops between β1–α2, β2–α3, β3–α4 and β4–α5. The AdoHcy adopts an *anti*- conformation, similar to that seen in the Ecm1, Hmc1 and ASFV MTase structures. Like most other RF MTases, these enzymes share a conserved, glycine-rich motif I (residues 58–62 in WBV-260) located in the β1–α2 loop, that accommodates AdoMet binding. In contrast, for the VV-D1 MTase the adenosine nucleoside is bound in the rarer *syn-*conformation, likely due to the side chain of an aspartic acid (D598), that clashes with the adenosine base in the *anti-*conformation (Supplementary Figure S3).

Several highly conserved residues contribute to the stabilization of the methyl donor in the active site (Figures 3A and 4A, B). The carboxylate group of AdoHcy is coordinated by K39 of motif X (located in α1), and its amine group is stabilized by a water molecule bond with D62 of motif I. D62 is located in the β1–α2 loop region, just downstream of the conserved glycine-rich motif. The ribose ring is stabilized by a hydrogen bond with the side chain of D77 of motif II. Finally, Y123 of motif III has a hydrophobic interaction with the ribose and purine group. Alanine mutagenesis of several conserved residues located around the AdoMet binding site, including K39, D77 and D54 completely abolish activity (Figure 3B). A D62A mutant was insoluble and thus could not be tested, suggesting that this residue also plays a critical role in the folding and stability of the domain. Mutation of the semi-conserved lysine in motif I (K59A) reduces activity to ∼20% compared to the wild-type enzyme. The residual activity suggests that it does not directly contribute to AdoMet binding, but rather may play a role in the methyltransfer reaction. Overall, these results show that the AdoMet binding pocket of the WBV N7-MT-1a is well conserved with other RF N7-MTases.

### Predicted cap binding site and coordination

The cap binding site can be inferred by the motif conservation (III, IV, V) and by analogy with the Ecm1 structure, complexed with ^m7^GpppG (PDB ID: 1RI2). The site is an open, moderately charged pocket, lined on one side by residues from α1 and α9 of the α-bundle (Figure 4A). The floor is formed by β4 and β5, topped by the 3β-flap domain. A hydrophobic pocket (Y240, F188, L152, P147, I146) ensures general positioning of the cap base of the GpppG, while guanine base selectivity is ensured by hydrogen bonding to conserved residues E180 (motif IV), H122 (motif III) and Y123 (motif V), whose side chain atoms contact the guanine O6, N1 or N2 atoms (Figure 4B, C). While an H122A mutant was insoluble and thus not tested, mutation of E180 and Y240 to alanine abolishes methyltransferase activity, confirming the importance of these residues for cap binding (Figure 3). The only mutant that retains a low level of activity is K59A, located in motif I. This residue is not predicted to perform direct contacts with the cap structure or AdoHcy, but likely aids in providing a positive electrostatic charge for the positioning of the triphosphate of the cap structure.

### The presence of the N7-MTase domain in non-mammalian infecting tobaniviruses

Using the newly defined WBV N7-MT-1a domain, we performed a MSA to look for the presence of this enzyme in other *Tobaniviridae* family members. This family is composed of 23 members which infect a range of mammalian (bovine, equine, porcine, etc.) and non-mammalian (various fish and reptiles) hosts. Remarkably, the N7-MT-1a domain appears to be present only in the 18 non-mammalian *Tobaniviridae* members (supplementary Figure 4). These viruses currently fall under the subfamilies *Serpentovirinae* and *Piscanivirinae*. While there is still some ambiguity for the precise location of N- and C-terminal domains, the characteristic secondary elements of RF enzymes (βaβ-fold) and sequence motifs could be easily identified for all of these viruses. We conclude that N7-MT-1a is a genetic marker for non-mammalian *Tobaniviridae*.

## DISCUSSION

Capping of viral RNA is a common process employed by many virus families to mimic the host eukaryotic mRNA, which is essential for RNA stability, translation, and innate-immune escape (7–10). The cytoplasmic lifestyle of these viruses requires them to carry their own capping machinery. Positive-sense RNA viruses vary considerably in their capping pathways and enzyme requirements (16). With the exception of the large-genome Nidoviruses, most +RNA viruses carry either a single methyltransferase domain, or none at all. For example, viruses of the *Picornaviridae* and *Caliciviridae* families do not possess any capping enzymes, but rather covalently attach a small viral protein (known as the VpG) to the 5′ end of the genome which functions as a cap-substitute (58). In the case of alphaviruses, the nsp1 protein harbors both GTase and MTase activity, first mediating the N7- methylation of GTP and subsequent transfer to RNA for formation of a cap-*O* structure (m^7^GpppN) (59). In contrast the Flavivirus MTase on NS5 is able to perform both N7- and 2′-*O* methylation of the RNA cap (54,60–64). Genome size and structural constraints for these viruses apparently meet the activity level requirements to process the single capped RNA species, that is, their (+)RNA genomes. The cap methylation demand for large-genome nidoviruses is thus probably much greater, and may have driven the evolutionary acquisition of two separate MTases to cope for high methylation needs on the numerous mRNA species (genomic and subgenomic RNAs).

For *Coroniviridae*, the cap *N*7- and 2′*O*- methyltransferases have been well-characterized, residing in nsp14 and nsp16 of pp1ab, respectively (5,19–21). Nsp16, located at the very end of ORF1b, is easily discernible as a 2′*O* MTase due to its K–D–K–E catalytic sequence motif and conserved RF structure. A homologous 2′*O* MTase domain can be mapped at the same genomic location for most of the other families in the *Nidovirales* order, with the exception of several small-genome nidoviruses, including the *Arteriviridae* family. In contrast, the identification of the N7-MTase domain has proved more challenging, and was seemingly lacking for several nidovirus families. In the CoV family, the N7-MTase domain has a distinct, non-RF structure (5). In most other nidoviral families however, this N7-MTase located at the nsp14 locus appears to be either completely absent, or its presence is uncertain due to lack of homology. Here, we confirmed the presence of a Class-1, N7-specific MTase in pp1a of WBV, a member of the *Tobaniviridae* family. The WBV N7-MT-1a exhibits a canonical RF βαβ structural motif, similar to that of diverse N7-MTases, including that of *E. cuniculi*, humans and two large dsDNA viruses, despite low sequence similarity. This proved challenging for molecular replacement, with all sequence based structural predictions (with the exception of AlphaFold2) failing to converge toward either a consensus model or a partial model suitable for phasing the data. In terms of sequence, structure and substrate-specificity, there is no homologous MTase currently identified for any other +RNA virus to date. It is also remarkably different from the ORF1b N7-MTase gene of CoVs, suggesting that these MTases were acquired through separate evolutionary events.

Using the newly defined WBV N7-MT-1a domain, we revisited the *Tobaniviridae* family. Remarkably, this domain is only present in the 18 non-mammalian host *Tobaniviridae* members. The acquisition of such a critical gene is a considerably rare event. Combined with its unexpected genomic location in ORF1a, we believe that the presence of this MTase is significant enough to warrant its consideration as a genetic marker for this family of viruses. Consequently, we propose that the other four mammalian-infecting Tobanivirus members be reassigned to a new, separate nidovirus family.

The genomic location of the N7-MTase in ORF1a is surprising, and demonstrates the plasticity of nidovirus genomes (Supplementary Figure S5). In general, the organization of genes encoded on ORF1a and ORF1b can be summarized as follows: ORF1a codes for scaffolding proteins for the replication complex, protein activators (protease and regulatory cofactors), and enzymes involved in antagonizing the innate immune system; ORF1b codes for RNA synthesis, editing or degradation enzymes, including nucleotide transferase, polymerase, NTPase-helicase, nucleases and methyltransferases (65). This division of functional tasks is likely used to regulate the copy numbers of specific genes. The proteins expressed from ORF1a are expected to be present at higher concentrations compared to those from ORF1b, whose expression is controlled by a −1 ribosomal frameshift. This raises the following questions: what is the benefit (or requirement) for these tobaniviruses to express higher levels of their N7-MTase? Furthermore, why is the same not true for the 2′*O* MTase, which maintains its conserved genomic location at the end of ORF1b? While we cannot definitively answer these questions, we speculate that it is related to the reptilian/fish host specificity of these viruses. More specifically, it has been shown that there is an inverse correlation between levels of DNA methylation and body temperature. In reptiles and fishes (cold-blooded animals), methylation levels are higher than those of warm-blooded mammals (66,67). It is therefore tempting to hypothesize that similar processes are at play regarding viral RNA cap methylation. Early and efficient methylation of the cap structure may be critical for escape from cellular immune sensors and for RNA stability. The acquisition of an N7-MTase gene in ORF1a is therefore in accordance with higher expression levels early during the viral replication cycle.

Alternatively, the tobanivirus N7-MT-1a may additionally recognize and methylate other substrates (distinct from the 5′ cap), potentially explaining the need for higher copy numbers of this enzyme. Both cellular and viral mRNAs have been shown to be internally methylated, most commonly at the m^6^A, m^5^C, m^1^G and ribose 2′-*O*-positions. In the case of viral RNA, these modifications may have either pro- or anti-viral effects, and may be involved in regulating viral replication, gene expression, and/or overcoming the antiviral response (7–10). Here, we did not observe internal methylation of short, synthetic RNAs in biochemical assays. While it is still possible that the WBV MTase recognizes a specific sequence or secondary-structure in the viral RNA for methylation, this seems unlikely from a functional point of view, given the specificity for methylation at the N7- position of the cap guanine.

Cellular MTases have additionally been shown to methylate various other RNA substrates, including transfer RNA (tRNA) at various bases and positions (68). A structural comparison of the WBV N7-MT-1a with class I, RF tRNA N-MTases shows distinct RF topologies, including the lack of 3β-flap and α-helical bundle domains, which would otherwise overlap the tRNA substrate binding-site (Supplementary Figure S6) (69). Methylation is also not limited to RNA substrates, with protein methyltransferases (PMTs) able to perform N-methylation of arginine and lysine side-chains, most commonly during histone modification (70). Of note, one of these PMTs was identified as the best HH-search hit for N7-MT-1a of two tobaniviruses, including WBV (30). While putative functions of this domain have been proposed based on sequence similarity alone (29,30), the statistical nature of the previous work stands as a prediction until demonstrated or disproved experimentally. The structure presented here clearly shows that the PMT structure is highly distinct from that of WBV (71) reflecting the critical adaptation of the catalytic site and confirming that the WB MTase structure is unambiguously an RNA MTase, as supported by the biochemical data.

Therefore, while we cannot definitively rule out the possibility that the tobanivirus N7-MT-1a recognizes another RNA substrate, the lack of a N7-MTase in ORF1b, the structural homology with known N7-guanine RNA MTases, the absence of homology with other protein or tRNA MTases and the strong N7-guanine specific cap activity, substantiates that the WBV pp1a MTase is the missing N7-guanine MTase involved in the RNA capping pathway.

In conclusion, we have demonstrated the presence of an N7-specific MTase domain in ORF1a of non-mammalian tobaniviruses, highlighting the amazing plasticity of the *Nidovirales* genome. Remarkably, this MTase is different from the ORF1b N7-MTase present in *Coronaviridae*. In the group of large RNA viruses, these two MTases were therefore likely acquired during separate events. Its structure belongs to the Rossman-fold family of enzymes, making it the first N7-specific RF MTase identified for (+)RNA viruses. Furthermore, its genomic location in ORF1a may also reflect a necessity for higher levels of methylation in cold-blooded hosts, supported by the fact that this domain is missing in tobaniviruses infecting mammalian hosts. Based on this, we propose that the *Tobaniviridae* family be further refined using this enzyme as a genetic marker.

## DATA AVAILABILITY

Reference alignment corresponding to *Tobaniviridae* N7-MT-1a domain was deposited to UniProt to update accession numbers polyprotein 1a (Q008X5) or 1a-1b (Q008X6).

Atomic coordinates and structure factors for the reported crystal structures have been deposited with the Protein Data bank under accession number 7Z05 and 7Z2J.

## Supplementary Material

gkac876_Supplemental_File
