# Supplementary material for: A second type of N7-guanine RNA cap methyltransferase in an unusual locus of a large RNA virus genome
Source: Nucleic Acids Res. 2022 Oct 21;50(19):11186–98. doi: 10.1093/nar/gkac876 (PMC9638943; doi:10.1093/nar/gkac876)
Supplement: gkac876_Supplemental_File [file gkac876_supplemental_file.pdf]

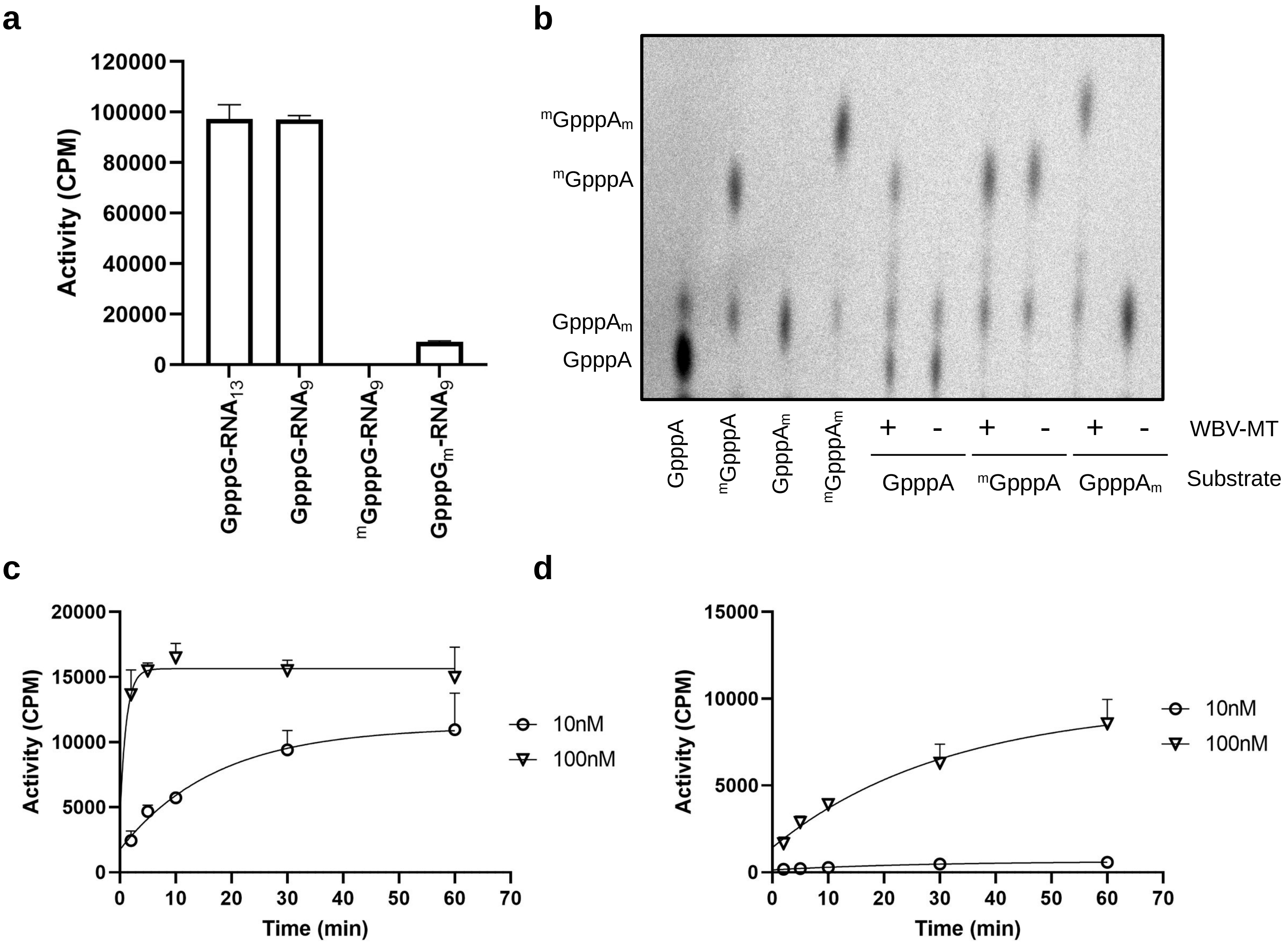

Supplementary Figure 1. Cap-dependent methyltransferase activity on different substrates. a) The transfer of tritiated methyl groups from AdoMet to different capped GpppG-RNAs (Table S1) was measured through filter binding assays and shown as counts per minute (CPM). Activity represents single, 30 minute timepoint measured for the WBV-295 product, and is shown as the mean ± SD (n=3). b) Thin-layer chromatography analysis showing migration of differently methylated GpppA cap structures. The first four lanes show migration of cap controls, prepared with the commercial, vaccinia virus N7-MTase. The remainder of gel shows modification of different capped RNAs (substrate) in the presence (+) or absence (-) of the WBV N7-MT-1a. c and d) Kinetics of FBA showing the transfer of tritiated methyl groups from AdoMet to GpppG-RNA_{13} (c) and GpppGm-RNA_{13} (d) at final enzyme concentrations of 10 and 100 nM (n=3, SD shown).

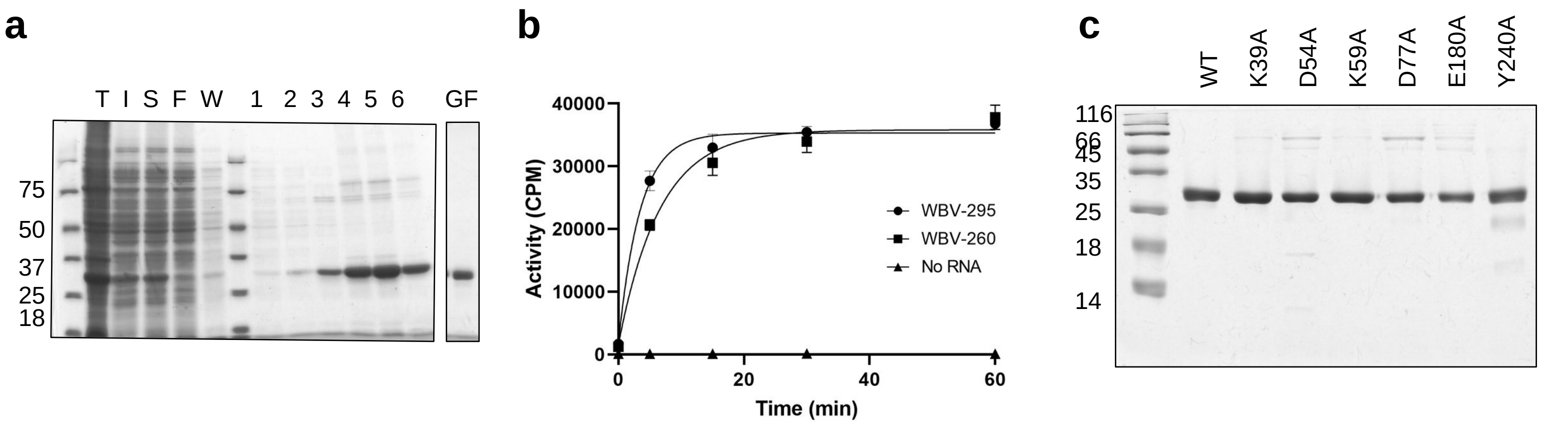

Supplementary Figure 2. Purification and activity of C-terminally truncated WBV N7-MT-1a domain. a) The WBV-295 product was C-terminally trunated to produce WBV-260, 30.5 kDa. Protein was expressed with N-terminal MRGSHHHHHH tag. Total fraction prior to cell lysis (T), Insoluble (I) and soluble (S) fractions following sonication and clarification, flow-through after nickle binding (F), wash (W), elutions (1-6), and gel-filtration product (GF) shown. b) Timecourse comparing the activity of WBV-295, and its C-terminally truncated version, WBV-260. The transfer of tritiated methyl groups from AdoMet to GpppG-RNA13 was measured through filter binding assays and shown as counts per minute (CPM). c) GF purified expression products of various WBV-260 mutants.

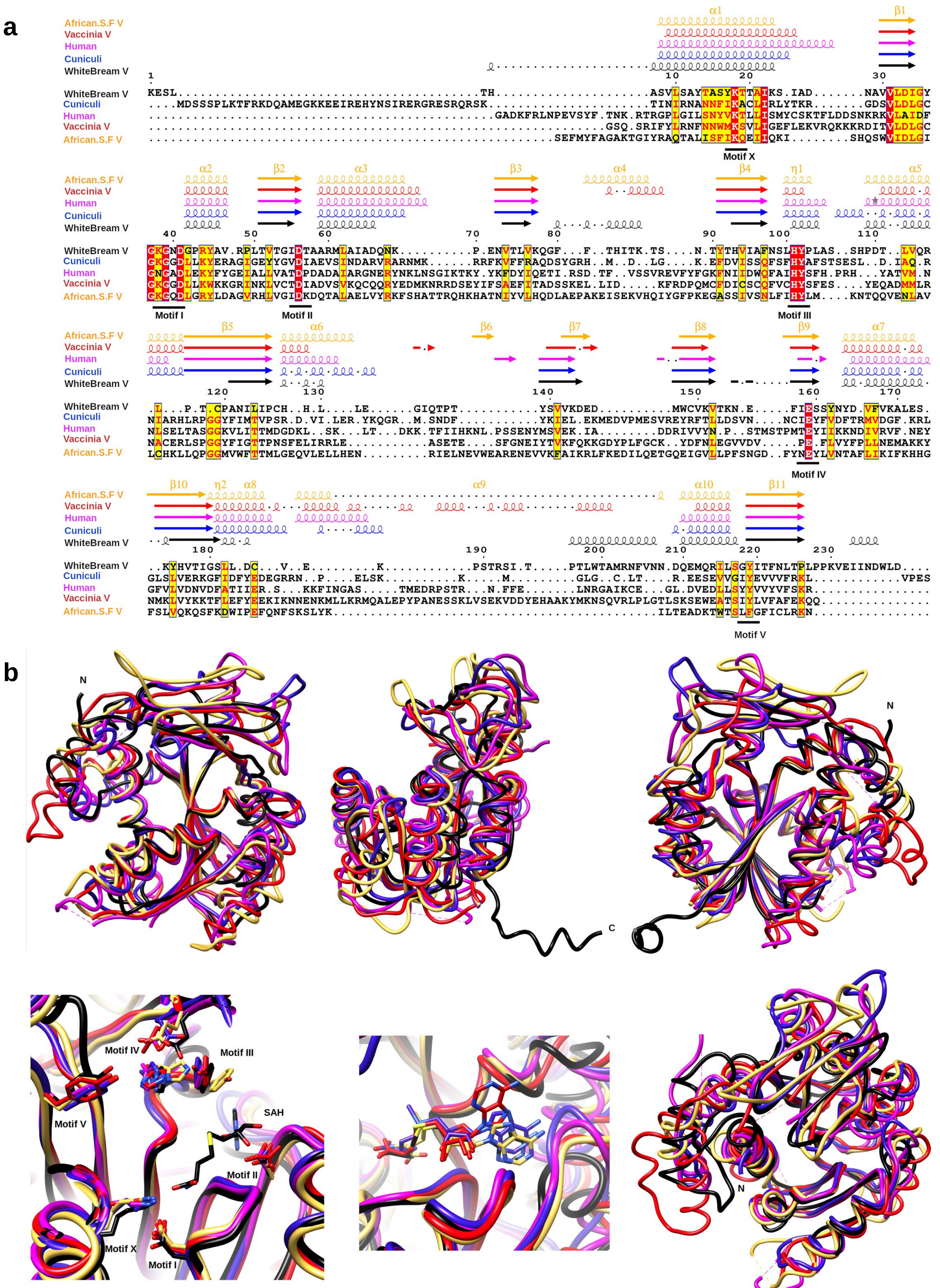

Supplementary Figure 3. a) Sequences alignment with secondary structure information derived from the structural superimposition of N7-MTase structures homologous to White Bream (PDB: 7Z05, black); ASFV (PDB: 7D8U, orange), Ecm1 (PDB: 1Z3C, blue), VV-D1 (PDB: 4CKB,red) and Hcm1 (PDB: 5E8J, pink). b) Structural superimposition of the above mentioned proteins. Color code is the same as in (a).

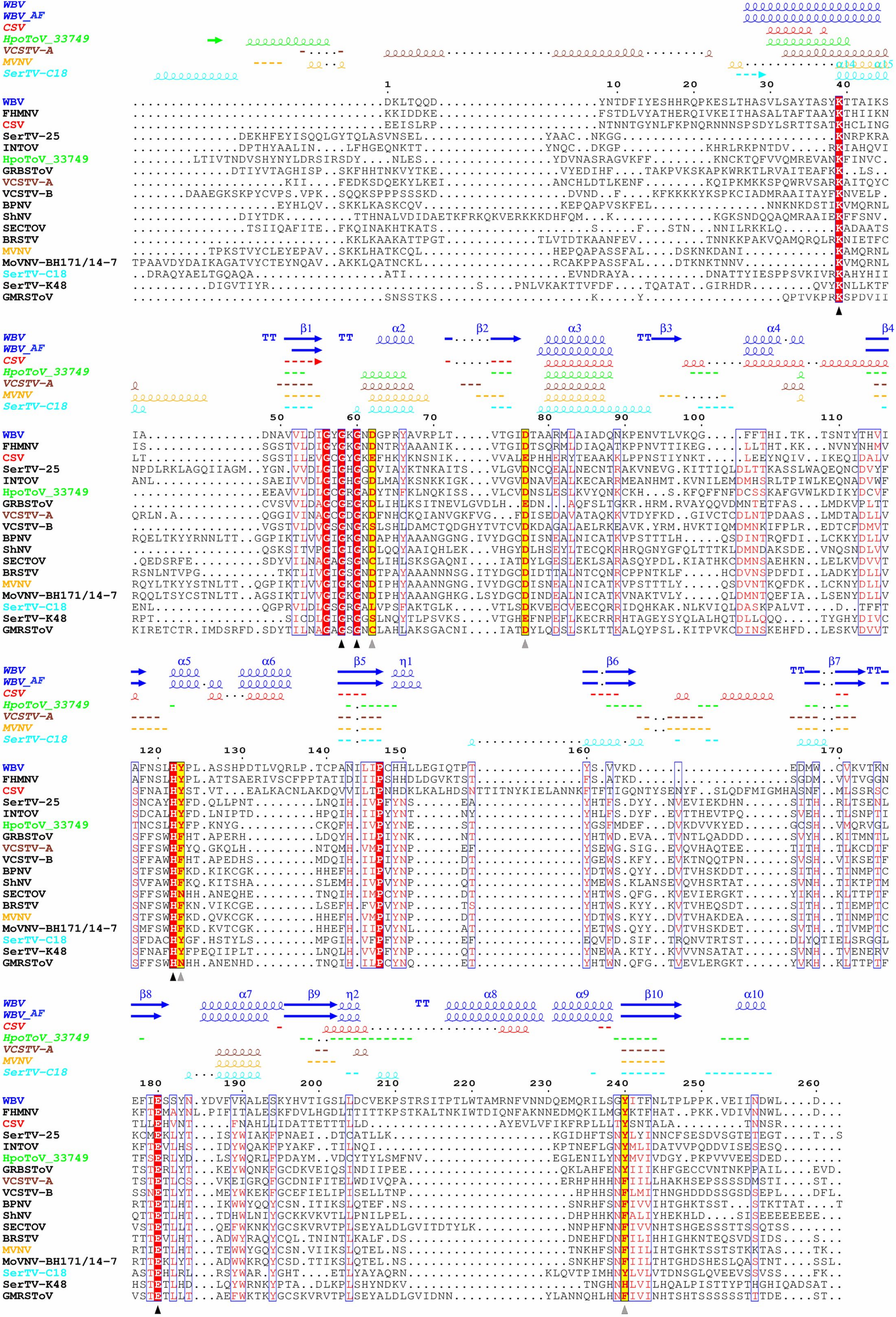

Supplementary Figure 4. Multiple sequence alignment of non-mammalian infecting *Tobaniviridae* N7-MT-1a domain showing secondary elements for WBV, and predicted secondary elements for original AlphaFold2 WBV prediction (WBV_AF), along with CSV, HpoToV_33749, VCSTV-A, MVNV and SerTV-C18.

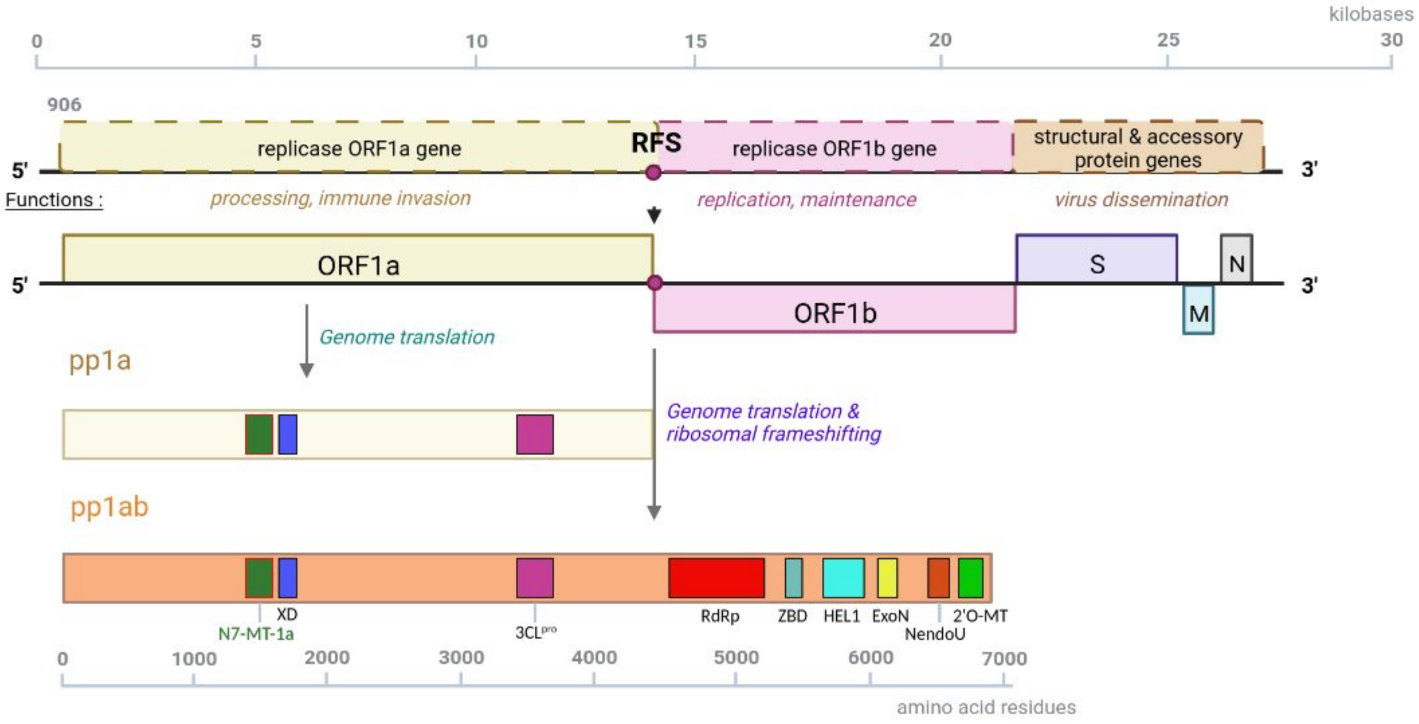

Supplementary Figure 5. BioRender figure representing the domain organization of the WBV genome. Top half of figure shows the two large overlapping ORFs; ORF1a & ORF1b, followed by structural protein genes; S,M & N. The bottom half of the figure represents the domain organization of the pp1a and pp1ab replicase polyproteins, the latter being produced by a -1 ribosomal frameshift. The approximative location of N7-MT-1a is shown in green in pp1a, and pp1ab. NCBI entry NC_008516.1 and UniProt entry Q008X6·R1AB_WBV24 were used as references to prepare this figure. Abbreviations – ORF, open reading frames; RFS, ribosomal frameshift signal; S, Spike; M, Membrane; N, Nucleocapsid; pp, polyprotein; N7-MT-1a, N7-guanine methyl transferase encoded in ORF1a; XD, Macro domain; 3CLpro, 3C-like or main protease; RdRp, RNA dependent RNA polymerase; ZBD, zinc-binding domain; HEL1, superfamily 1 helicase; ExoN, 3'-to-5' exoribonuclease; NendoU, uridylate-specific endoribonulease; 2'O-MT, 2'-O-methyl transferase.

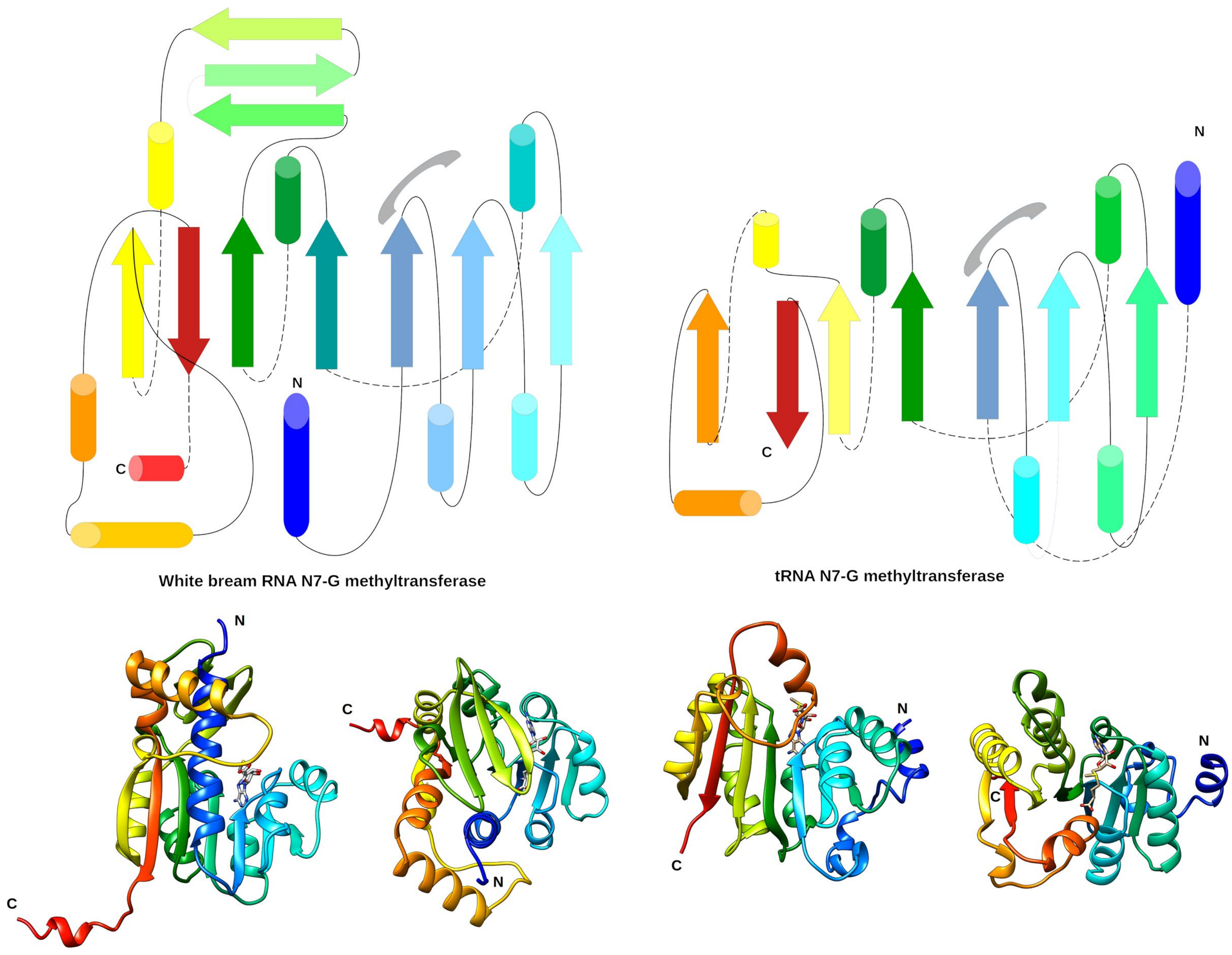

Supplementary Figure 6. Topology of WBV N7-MT-1a (top left) and tRNA N7-G methyltransferase (top right), with structures shown below. Position of SAM binding-site indicated with grey semicircle.

Supplementary Table 1. List of RNA sequences used for enzymatic characterization

Name	Sequence and methylation status
**GpppG-RNA$_{13}$**	GpppGAUGAAGAUUAAG
**mGpppG-RNA$_{13}$**	mGpppGAUGAAGAUUAAG
**GpppGm-RNA$_{13}$**	GpppGmAUGAAGAUUAAG
**mGpppGm-RNA$_{13}$**	mGpppGmAUGAAGAUUAAG
**GpppG(Nm)-RNA$_{13}$**	GpppGAmUmGmAmAmGmAmUmUmAmAmGm
**mGpppG(Nm)-RNA$_{13}$**	mGpppGAmUmGmAmAmGmAmUmUmAmAmGm
**GpppGm(Nm)-RNA$_{13}$**	GpppGmAmUmGmAmAmGmAmUmUmAmAmGm
**GpppA(Am)-RNA$_{13}$**	GpppAUGAmUGAmAmGAmUUAm
**GpppG-RNA$_{9}$**	GpppGGGACAAGU
**mGpppG-RNA$_{9}$**	mGpppGGGACAAGU
**GpppGm-RNA$_{9}$**	GpppGmGGACAAGU

Supplementary Table 2. List of the *Tobaniviridae* sequences used in the analysis

S.No.	Virus name	Species	Abbreviation	Accession No.	Classification - Subfamily
1	White bream virus	*White bream virus*	WBV	NC_008516	*Piscanivirinae*
2	Fathead minnow nidovirus	*Fathead minnow nidovirus 1*	FHMNV	NC_038295	*Piscanivirinae*
3	Chinook salmon bafinivirus isolate NIDO	*Chinook salmon nidovirus 1*	CSV	NC_026812	*Piscanivirinae*
4	Serpentovirinae sp. isolate L25	*Infratovirus latu*	SerTV-25	MN161572	*Serpentovirinae*
5	Xinzhou toro-like virus strain XZSJSC65757	*Infratovirus 1*	INTOV	NC_033700	*Serpentovirinae*
6	Hainan hebius popei torovirus LPSC33749	*Hebius tobanivirus 1*	HpoToV_33749	MG600028	*Serpentovirinae*
7	Guangdong red-banded snake (Lycodon rufozonatus) torovirus LPSF30546	*Lycodon tobanivirus 1*	GRBSToV	MG600030	*Serpentovirinae*
8	Veiled chameleon serpentovirus A	*Lyctovirus alpa*	VCSTV-A	MT997160	*Serpentovirinae*
9	Veiled chameleon serpentovirus B	*Vebetovirus paba*	VCSTV-B	MT997159	*Serpentovirinae*
10	Ball python nidovirus strain 07-53	*Ball python nidovirus 1*	BPNV	NC_024709	*Serpentovirinae*
11	Shingleback nidovirus 1	*Shingleback nidovirus 1*	ShNV	KX184715	*Serpentovirinae*
12	Xinzhou nematode virus 6	*Sectovirus 1*	SECTOV	NC_043490	*Serpentovirinae*
13	Bellinger River snapping turtle virus J248	*Berisnavirus 1*	BRSTV	MF685025	*Serpentovirinae*
14	Morelia viridis nidovirus strain S14-1323_MVNV	*Morelia tobanivirus 1*	MVNV	NC_035465	*Serpentovirinae*
15	Morelia viridis nidovirus isolate BH171/14-7	*Pregotovirus heba*	MoVNV-BH171/14-7	MK182569	*Serpentovirinae*
16	Serpentovirinae sp. isolate C18	*Sertovirus cona*	SerTV-C18	MN161561	*Serpentovirinae*
17	Serpentovirinae sp. isolate K48	*Septovirus foka*	SerTV-K48	MN161566	*Serpentovirinae*
18	Guangdong mandarin rat snake torovirus LPSF32319	*Sectovirus 2*	GMRSToV	MG600031	*Serpentovirinae*
19	Bovine nidovirus TCH5	*Bovine nidovirus 1*	BoNV	NC_027199	*Remotovirinae*
20	Berne virus isolate P138/72	*Equine torovirus*	EToV	MG996765	*Torovirinae*
21	Porcine torovirus strain SH1	*Porcine torovirus*	PoTV	NC_022787	*Torovirinae*
22	Bangali torovirus	*Torovirus banli*	BaToV-Hrufipes2018	MW561977	*Torovirinae*
23	Breda virus	*Bovine torovirus*	BRV	NC_007447	*Torovirinae*
